# Renin-angiotensin system modulators and other risk factors in COVID-19 patients with hypertension: a Korean perspective

**DOI:** 10.1186/s12879-021-05848-8

**Published:** 2021-02-15

**Authors:** Hee-Sung Kim, Minseok Kang, Gilwon Kang

**Affiliations:** 1 Department of Internal Medicine, Chungbuk National University College of Medicine, Cheongju, Republic of Korea; 2 Department of Health Information and Management, Chungbuk National University College of Medicine, 1 Chungdae-ro, Seowon-gu, Cheongju, 28644 Republic of Korea; 3 Chungbuk Regional Cardiovascular Center, Chungbuk National University Hospital, Cheongju, Republic of Korea

**Keywords:** COVID-19, Hypertension, Angiotensin receptor blocker (ARB), Angiotensin converting enzyme inhibitor (ACEI)

## Abstract

**Background:**

While hypertension is the most common comorbid condition in patients with coronavirus disease 2019 (COVID-19) in Korea, there is a lack of studies investigating risk factors in COVID-19 patients with hypertension in Korea. In this study, we aimed to examine the effects risk factors in hypertensive Korean COVID-19 patients.

**Methods:**

We selected patients from the database of the project #OpenData4Covid19. This information was linked to their 3-year historical healthcare data. The severity of the disease was classified into five levels. We also clustered the levels into two grades.

**Results:**

The risk factors associated with COVID-19 severity were old age, diabetes mellitus, cerebrovascular disease, chronic obstructive pulmonary disease (COPD), malignancy, and renal replacement therapy. The use of angiotensin converting enzyme inhibitors (ACEIs) or angiotensin receptor blockers (ARBs) both before and after a diagnosis of COVID-19 were not associated with COVID-19 severity. A multivariate analysis revealed that old age, male sex, diabetes mellitus, and renal replacement therapy were risk factors for severe COVID-19.

**Conclusion:**

The results suggest that in hypertensive patients with COVID-19, older age, male sex, a diagnosis of diabetes mellitus, and renal replacement therapy were risk factors for a severe clinical course. In addition, the use of ARBs and ACEIs before or after COVID-19 infection did not affect a patient’s risk of contracting COVID-19 nor did it contribute to a worse prognosis for the disease. These results highlighted that precautions should be considered for hypertensive patients with those risk factors and do not support discontinuation of ARBs and ACEIs during COVID-19 pandemic.

## Background

Among laboratory-confirmed cases of coronavirus disease (COVID-19), patients with underlying disease such as hypertension, diabetes, cardiovascular disease, respiratory disease, and malignancy have a poorer clinical outcome [1, 2]. In 2016, the percentage of the Korean population that was diagnosed with hypertension was 29.1% [3]. Due to its high prevalence, hypertension is the condition most comorbid with COVID-19 in Korea [4]. While hypertension is a known prognostic indicator of disease severity and mortality in COVID 19, it is not clear whether this link is due to the actual hypertensive condition itself, the presence of other comorbidities, or the type of anti-hypertensive treatment regimen being followed^.^

There is also a debate concerning the role played by the angiotensin-converting enzyme 2 (ACE2) in the pathogenesis of COVID 19; angiotensin receptor blockers are the most frequently used monotherapy drugs in Korea [5]. The debate centers around the effect of RAS modulators such as ACE inhibitors (ACEIs) and angiotensin receptor blockers (ARBs) on severe acute respiratory syndrome coronavirus-2 (SARS-CoV-2) infectivity. One school of thought is that these renin-angiotensin system (RAS) modulators increase the risk of developing severe COVID-19, since ACE2 facilitates the entry of SARS-CoV-2 into the cell [6]. Another school of thought is that these RAS modulators improve the clinical outcome of COVID-19 by regulating the immune function and attenuating the inflammatory response [7].

In this study, we assessed the effects of RAS modulators and other risk factors in COVID-19 patients with hypertension in Korea.

## Methods

### Patient selection and classification

**Fig. 1:**
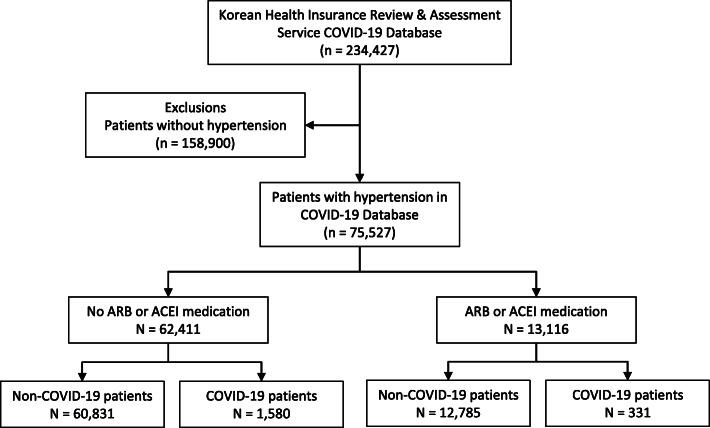
A flow-chart of patient selection

We selected patients selected from the database of the project #OpenData4Covid19, a global research collaboration on COVID-19, hosted jointly by the Ministry of Health and Welfare of Korea and the Health Insurance Review and Assessment (HIRA) Service. The database contained information on the insurance benefit claims sent to HIRA including the data of all the patients that claimed for a COVID-19 test. This information was linked to their 3-year historical healthcare data. From January 3 to May 15, 2020, 234,427 individuals were tested for COVID-19, and 75,527 were diagnosed with hypertension. Of the 13,116 individuals that were on RAS modulators, 331 had a laboratory-confirmed COVID-19 diagnosis. HIRA data base contains every confirmed COVID-19 cases since it containing COVID-19 case is generated in the process of reimbursing providers and people with a COVID-19 test with a negative result maintained this status (If this people take their retest with positive results, this people turned into confirmed case). Of the 62,411 individuals that were not on any RAS modulators, 1580 had a laboratory- confirmed COVID-19 diagnosis. Laboratory diagnosis of COVID-19 are being carried out by national central labs and 95 certified non-governmental clinical laboratories. Laboratory confirmed COVID-19 was defined by approved real-time PCR protocols targeting *E, RdRp, N, orf1b, orf1a* genes. For diagnostic testing for SARS-CoV-2, upper respiratory tract specimen and lower respiratory tract specimen were used. We excluded re-detected cases that test positive for SARS-CoV-2 after being discharged from isolation. Since it is considered that the genetic material of the “dead virus” remaining in a recovered patient’s body is detected. A flow-chart of patient selection is presented in Fig. 1. This study was approved by the Institutional Review Board of Chungbuk National University Hospital (2020–04–015-001).

We merged the COVID-19 medical insurance claim data with the patient data and excluded cases of COVID-19 re-infection. The severity of the disease was classified into five levels: mild, moderate, severe, critical, and death. Each level of severity was defined according to the health insurance procedure code. Mild cases were defined by the lack of need for oxygen, moderate cases required oxygen therapy (M0040), severe cases required mechanical ventilation (MV) (M0850, M0857, M0858, M0860, M5830, M5850, M5857, M5858, M5860), and critical cases required extracorporeal membrane oxygenation (ECMO) (O1903, O1904). We also clustered the levels into two grades. Moderate, severe, critical, and death levels were clustered as Severity grade 1, while severe, critical, and death levels were clustered as Severity grade 2.

Comorbidities was identified using the International Classification of Disease, 10th revision (ICD-10) codes: hypertension I10, diabetes mellitus E10-E14, cardiovascular disease (I11-I13), cerebrovascular disease (I60-I69), ischemic heart disease (I20-I25), and chronic obstructive pulmonary disease (J440, J441, J448, J449).

We used expanded benefit coverage codes or specific exemption codes for rare incurable diseases to identify malignancy (V193) and renal replacement therapy (V001, V003, V005). The use of ACEI and ARB was identified by their specific ATC code: angiotensin-converting enzyme (ACE) inhibitors (C09AA, C09BA, C09BB, C09Bx) and angiotensin-receptor II blockers (C09CA, C09DA, C09DB, C09DX).

We defined exposure to antihypertensive medication before diagnosis of COVID-19 as at least one prescription of antihypertensive medication from January 1, 2020 until the diagnosis of COVID-19. This information was obtained from inpatient and outpatient prescription records of antihypertensive medication.

### Statistical analyses

In the general characteristics of the analysis data, the subjects of hypertension and non-hypertension were classified, and the mean and median values for the continuous variable and the frequency and ratio for the categorical variable were indicated. In hypertensive patients, a chi-square test was performed for each independent variable, frequency and ratio, which are expected to affect COVID-19 infection risk. In addition, multivariate logistic regression was performed to confirm the influence of the independent variable considering the interaction of each variable. The severity of COVID-19 patients was divided into five stages, and the independent variables, frequencies and ratios expected to affect clinical severity were analyzed by performing a chi-square test. Multivariate analysis was performed using logistic regression to evaluate the association between selected clinical characteristics and a likelihood of a positive test for COVID-19/COVID-19 severity. SAS Enterprise Guide Software version 6.1 (SAS Institute Inc., Cary, NC) was used for these analyses, and a *P* value of less than .05 was considered statistically significant.

## Results

Table 1 reports the characteristics of the study population of patients tested for COVID-19. Until 15th May, 2020, a total of 234,427 patients were tested for COVID-19 and 7590 (3.2%) had a positive result. Among the patients tested for COVID-19, 75,527 (32.2%) had a history of hypertension; of these, 1911(2.5%) were COVID-19-positive and 13,116 (17.4%) patients took ACE inhibitors or ARBs. Patients with hypertension were likely to be older and have more comorbidities than those without.

**Table 1:** Characteristics of the study population of patients tested for COVID-19

Characteristics	Patients with hypertension (***N*** = 75,527)	All patients (***N*** = 234,427)
Median age (interquartile rage)- yr.	68 (23)	45 (35)
Female sex – no. (%)	35,761 (47.3)	122,480 (52.2)
Confirmed COVID-19- no. (%)	1911 (2.5)	7590 (3.2)
Diabetes mellitus- no. (%)	44,441 (58.8)	62,655 (26.7)
Cardiovascular disease- no. (%)	4705 (6.2)	5038 (2.1)
Cerebrovascular disease- no. (%)	14,503 (19.2)	17,519 (7.5)
Ischemic heart disease- no. (%)	12,122 (16)	14,191 (6.1)
COPD- no. (%)	4871 (6.4)	6612 (2.8)
Malignancy- no. (%)	14,368 (19)	24,628 (10.5)
Renal replacement therapy- no. (%)	4338 (5.7)	4525 (1.9)
ACEIs or ARBs- no. (%)	13,116 (17.4)	13,240 (5.6)

Compared to patients who tested negative for COVID-19, those that tested positive were more likely to be younger (OR: 80.67) and women (OR 56.63) (Table 2). COVID-19 was confirmed more frequently in people without comorbidities, such as diabetes mellitus, cerebrovascular diseases, ischemic heart disease, COPD, malignancy, and renal replacement therapy. Use of ACEI or ARB was not different between two groups.

**Table 2:** Characteristics of the patients with hypertension tested for COVID-19

	COVID-19 (−)	COVID-19 (+)	Difference (95% CI)
**Age**			80.6791***
< 60	22,850 (0.97)	665 (0.03)	
61–69	15,609 (0.97)	514 (0.03)	
70–79	17,899 (0.98)	418 (0.02)	
≥ 80	17,258 (0.98)	314 (0.02)	
**Sex**			56.6345***
Male	38,922 (0.98)	844 (0.02)	
Female	34,694 (0.97)	1067 (0.03)	
**Diabetes Mellitus**			29.5498***
Yes	30,184 (0.97)	902 (0.03)	
No	43,432 (0.98)	1009 (0.02)	
**Cardiovascular disease**			2.9933
Yes	69,012 (0.97)	1810 (0.03)	
No	4604 (0.98)	101 (0.02)	
**Cerebrovascular disease**			26.7711***
Yes	59,392 (0.97)	1632 (0.03)	
No	14,224 (0.98)	279 (0.02)	
**Ischemic heart disease**			52.4329***
Yes	61,686 (0.97)	1719 (0.03)	
No	11,930 (0.98)	192 (0.02)	
**COPD**			53.0971***
Yes	68,791 (0.97)	1865 (0.03)	
No	4825 (0.99)	46 (0.01)	
**Malignancy**			180.4433***
Yes	59,384 (0.97)	1775 (0.03)	
No	14,232 (0.99)	136 (0.01)	
**Renal replacement therapy**			74.6484***
Yes	69,301 (0.97)	1888 (0.03)	
No	4315 (0.99)	23 (0.01)	
**ACEIs or ARBs**			0.0028
Yes	12,785 (0.97)	331 (0.03)	
No	60,831 (0.97)	1580 (0.03)	

* ***P*** < .05; ** ***P*** < .01; *** ***P*** < .001

Multivariate analysis revealed that laboratory confirmed cases were less prevalent in males and individuals with cerebrovascular disease, ischemic heart disease, chronic obstructive pulmonary disease, malignancy, and renal replacement therapy (Table 3). Compared to the < 60 years age group, confirmed COVID-19 cases were more frequent in the 61–69 years age group; however, they were less frequent in the ≥80 years age group.

**Table 3:** A multivariate analysis of a likelihood of a positive test for COVID-19

	COVID − 19 (+)	Difference (95% CI)
ACEIs or ARBs	1.01 (0.89–1.13)	0.9345
61–69	1.36 (1.2–1.53)	<.0001
70–79	1.03 (0.91–1.18)	0.3633
≥80	0.7 (0.6–0.8)	<.0001
Female	1.33 (1.21–1.46)	<.0001
Diabetes mellitus	0.92 (0.84–1.01)	0.0739
Cardiovascular disease	0.89 (0.73–1.09)	0.2599
Cerebrovascular disease	0.76 (0.67–0.87)	<.0001
Ischemic heart disease	0.65 (0.55–0.75)	<.0001
COPD	0.43 (0.32–0.58)	<.0001
Malignancy	0.31 (0.26–0.37)	<.0001
Renal replacement therapy	0.19 (0.13–0.29)	<.0001

* ***P*** < .05; ** ***P*** < .01; *** ***P*** < .001

Univariate analysis of risk factors associated with COVID-19 severity revealed that old age, diabetes mellitus, cerebrovascular disease, COPD, malignancy, and renal replacement therapy were significant risk factors for severe COVID-19 (Table 4). Use of ACEI or ARB before or after diagnosis of COVID-19 was not associated with the severity of COVID-19. Patients were grouped according to level of care, we analyzed risk factors between mild cases and severity grade 1 (moderate, severe, critical), mild or moderate cases and severity grade 2 (severe, critical) and death cases and others (mild, moderate, severe, critical). Multivariate analysis revealed that old age, male sex, diabetes mellitus, and renal replacement therapy were risk factors for severity of COVID-19 (Table 5).

**Table 4:** Risk factors associated with COVID-19 severity

	Room air	O_**2**_ supply	MV	ECMO	Death	Difference (95% CI)
**Age**						335.0999***
< 60	579 (0.87)	72 (0.11)	4 (0.01)	1 (0)	9 (0.01)	
61–69	363 (0.71)	115 (0.22)	10 (0.02)	3 (0.01)	23 (0.04)	
70–79	246 (0.59)	113 (0.27)	6 (0.01)	1 (0)	52 (0.12)	
≥ 80	134 (0.43)	78 (0.25)	4 (0.01)	1 (0)	97 (0.31)	
**Sex**						6.3159
male	562 (0.67)	174 (0.21)	13 (0.02)	3 (0)	92 (0.11)	
female	760 (0.71)	204 (0.19)	11 (0.01)	3 (0)	89 (0.08)	
**Diabetes mellitus**						40.4894***
No	683 (0.76)	151 (0.17)	11 (0.01)	3 (0)	54 (0.06)	
yes	639 (0.63)	227 (0.22)	13 (0.01)	3 (0)	127 (0.13)	
**Cardiovascular disease**						1.1868
No	1255 (0.69)	357 (0.2)	22 (0.01)	6 (0)	170 (0.09)	
Yes	67 (0.66)	21 (0.21)	2 (0.02)	0 (0)	11 (0.11)	
**Cerebrovascular disease**						24.6214***
No	1158 (0.71)	309 (0.19)	22 (0.01)	6 (0)	137 (0.08)	
Yes	164 (0.59)	69 (0.25)	2 (0.01)	0 (0)	44 (0.16)	
**Ischemic heart disease**						8.4676
No	1204 (0.7)	334 (0.19)	20 (0.01)	6 (0)	155 (0.09)	
Yes	118 (0.61)	44 (0.23)	4 (0.02)	0 (0)	26 (0.14)	
**COPD**						12.8381*
No	1300 (0.7)	363 (0.19)	24 (0.01)	6 (0)	172 (0.09)	
Yes	22 (0.48)	15 (0.33)	0 (0)	0 (0)	9 (0.2)	
**Malignancy**						11.4142*
No	1241 (0.7)	348 (0.2)	22 (0.01)	6 (0)	158 (0.09)	
Yes	81 (0.6)	30 (0.22)	2 (0.01)	0 (0)	23 (0.17)	
**Renal replacement therapy**						18.3735***
No	1315 (0.7)	367 (0.19)	24 (0.01)	6 (0)	176 (0.09)	
Yes	7 (0.3)	11 (0.48)	0 (0)	0 (0)	5 (0.22)	
**ARB/ACEI use before** **COVID-19 diagnosis**						0.5448
No	1094 (0.69)	310 (0.2)	19 (0.01)	5 (0)	152 (0.1)	
Yes	228 (0.69)	68 (0.21)	5 (0.02)	1 (0)	29 (0.09)	
**ARB/ACEI use after** **COVID-19 diagnosis**						8.7247
No	1259 (0.6)	355 (0.2)	21 (0.01)	5 (0)	177 (0.1)	
Yes	63 (0.67)	23 (0.24)	3 (0.03)	1 (0.01)	4 (0.04)	

* ***P*** < .05; ** ***P*** < .01; *** ***P*** < .001

**Table 5:** A multivariate analysis of risk factors for COVID-19 severity

	Severity grade 1*		Severity grade 2†		Death‡	
	Odds Ratio	***P***	Odds Ratio	***P***	Odds Ratio	***P***
ARB/ACEI use before COVID- 19 diagnosis	1.05 (0.79–1.39)	0.759	0.99 (0.65–1.51)	0.9556	1 (0.63–1.58)	0.9851
ARB/ACEI use after COVID- 19 diagnosis	1.35 (0.83–2.2)	0.2206	0.92 (0.41–2.03)	0.8279	0.5 (0.17–1.44)	0.1978
Age group: 61–69	2.89 (2.13–3.93)	0.0685	3.48 (1.84–6.58)	0.0175	3.38 (1.53–7.46)	0.0033
Age group: 70–79	4.76 (3.47–6.53)	0.0002	7.48 (4.05–13.83)	0.0045	9.99 (4.76–20.99)	0.0008
Age group: ≥80	9.69 (6.9–13.59)	<.0001	24.66 (13.48–45.13)	<.0001	35.89 (17.31–74.43)	<.0001
Female	0.66 (0.53–0.82)	0.0001	0.52 (0.38–0.72)	<.0001	0.51 (0.36–0.73)	0.0002
Diabetes mellitus	1.46 (1.17–1.81)	0.0007	1.63 (1.17–2.27)	0.0038	1.79 (1.24–2.57)	0.0018
Cardiovascular disease	0.98 (0.62–1.56)	0.9361	0.93 (0.48–1.78)	0.8206	0.88 (0.43–1.77)	0.7089
Cerebrovascular disease	1.16 (0.87–1.54)	0.3179	1.08 (0.73–1.58)	0.7028	1.23 (0.82–1.84)	0.3121
Ischemic heart disease	0.99 (0.71–1.38)	0.9361	0.97 (0.62–1.53)	0.8956	0.93 (0.57–1.52)	0.7828
COPD	1.44 (0.76–2.69)	0.2611	0.92 (0.41–2.05)	0.8334	1.04 (0.46–2.34)	0.9345
Malignancy	1.17 (0.79–1.73)	0.4253	1.36 (0.82–2.24)	0.2299	1.45 (0.85–2.44)	0.17
Renal replacement therapy	9.99 (3.82–26.1)	<.0001	4.13 (1.31–13.02)	0.0154	5.64 (1.71–18.58)	0.0045

* Severity grade 1(moderate, severe, critical and death cases) was defined as a more serious cases than mild cases. Severity grade 1 was compared with mild cases

† Severity grade 2(severe, critical and death cases) was defined as a more serious cases than moderate cases. Severity 2 grade was compared with mild or moderate cases

‡ Death cases were compared with mild, moderate, severe, critical cases

## Discussion

Being older and of the male sex have been described as risk factors for a highly severe disease course in patients with COVID-19 [4, 8]. In China, case fatality rate (CFR) for those ≥80 years of age was 14.8% and in Korea, it was 14%. The CFR was also much higher in regions with collapsed health care systems. A recent study showed that men had a higher case of fatality that was independent of age [8, 9] This finding is thought to be due reasons such as gender specific life behavior patterns or sex differences in immune responses [10, 11]. Another report from China indicated that ACE2 levels which are correlated with organ failure are higher in men than women [12]. Caution should be taken to treat COVID-19 patient with diabetes mellitus, since patients with diabetes mellitus have a poorer prognosis especially when metabolic complications of pre-existing diabetes are observed [13]. In our study of hypertensive patients with COVID-19, older age, male sex, a diagnosis of diabetes mellitus, and renal replacement therapy were risk factor for a more severe clinical prognosis for the disease.

While recent studies have implicated the presence of comorbidities as well as pro-inflammatory and pro-coagulative states in severe COVID-19 outcomes, SARS-CoV-2 itself also has a negative effect on beta cell functions, precipitating acute metabolic complications [14]. Patients on dialysis have depressed immune systems and usually have other comorbidities [15]. A meta-analysis has shown that chronic kidney disease (CKD) seems to be associated with an enhanced risk of severe COVID-19 infection [16]. These patients are also at a higher risk of contracting COVID-19, since the in-center hemodialysis units are often very densely populated. Our data indicated that patients on dialysis are at greater risk of severe COVID-19 infection.

There are conflicting reports on the effects of ARBs or ACEIs on the clinical results of patients with COVID-19. While clinical evidence indicates that these drugs can protect the lung from pneumonia and reduce SARS-CoV-2 -induced lung injury [17], other researchers recommend discontinuing their use on the grounds that their use may enhance the risk of COVID-19. This is based on experimental findings that ARBs upregulate ACE and may thus enhance viral uptake and increase its virulence [5].

SARS-CoV-2 uses the ACE2 receptor for entry into the cell, and there have been concerns about whether these RAS modulators can upregulate the ACE2 receptor and modify susceptibility to COVID-19 [18].

Current research has demonstrated that use of either ACEIs or ARBs does not increase the likelihood of a positive test, and experts recommend that ACEI and ARB treatment regimens not be withdrawn [19]. The prevailing consensus is that being on either an ARB or ACEI treatment protocol, is not associated with a higher risk of testing positive for COVID-19. After adjusting age, sex and comorbidities, our study confirms that the use of ARBs and ACEIs is not associated with a greater severity of COVID-19 and supports the view that patients on ACEI or ARB treatment regimens should continue their medication as prescribed.

Our study has four limitations. First, the findings cannot be generalized to the general population due to the inhomogeneity of the study population. From February 18 until May 15, 2020 the large number of COVID-19 cases in Korea stemmed from a religious group in the Daegu and Gyeongbuk provinces and we were not able to correlate data regarding religion, contact with a confirmed case, and real area of residence. Therefore, in our study, the COVID-19 exposure risk and the susceptibility to COVID-19 may be biased. Second, we were not able to analyze laboratory data and data regarding antiviral and steroid usage. Third, the severity of cases was identified and analyzed for all cases. Therefore, the level of severity may have been underestimated, because many of the open cases could end in death. Fourth, actual drug exposure to RAS modulators could not be quantified since the electronic health data did not include detailed data regarding patient compliance and the dose of the medication used.

## Conclusions

In conclusion, we found that in hypertensive patients with COVID-19, older age, male sex, a diagnosis of diabetes mellitus, and renal replacement therapy were risk factors for a severe clinical course. In addition, the use of ARBs and ACEIs before or after COVID-19 infection did not affect a patient’s risk of contracting COVID-19 nor did it contribute to a worse prognosis. To prioritize at-risk populations and allocate resource, precautions should be considered for hypertensive patients with those risk factors and our data do not support discontinuation of ARBs and ACEIs during COVID-19 pandemic.

## Data Availability

The claim data provided by Health Insurance Review and Assessment Service are not publicly available since HIRA put an end to the collaboration project (#OpenData4Covid19) on July 31, 2020. Thus, we cannot share the data we used for this study with other researchers.

## Abbreviations

ACEIs: Angiotensin Converting Enzyme Inhibitors
ARBs: Angiotensin Receptor Blockers
ACE2: Angiotensin Converting Enzyme 2
COVID-19: Coronavirus Disease 2019
COPD: Chronic obstructive pulmonary disease
CFR: Case fatality rate
CKD: Chronic kidney disease
ECMO: Extracorporeal membrane oxygenation
HIRA: Health Insurance Review and Assessment
ICD: International Classification of Disease
MV: Mechanical ventilation
RAS: Renin-Angiotensin System
SARS-CoV-2: Severe Acute Respiratory Syndrome Coronavirus-2
